# Synergistic Effects of Selenium, Selenoproteins, Selenocysteine and Procyanidin A2 Based on Immune Response and Oxidative Stress

**DOI:** 10.3390/molecules31142503

**Published:** 2026-07-17

**Authors:** Liang Yang, Yunyun Ma, Xuan Wu, Jinnan Sun, Rong Qian, Yaoxiang Liang, Bin Zheng, Jinmiao Zhu

**Affiliations:** 1 School of Chemistry and Pharmaceutical Engineering, Hefei Normal University, Hefei 230601, China; yangliang@hfnu.edu.cn (L.Y.);; 2 Key Laboratory of Modern Preparation of TCM, Ministry of Education, Jiangxi University of Traditional Chinese Medicine, Nanchang 330004, China

**Keywords:** procyanidin, selenocysteine, tumor, synergistic effect

## Abstract

Proanthocyanidin A_2_ (PA_2_) is a class of bioactive small-molecule natural polyphenols, while selenocysteine (Sec) represents an essential functional selenium-containing amino acid in humans and has emerged as a cutting-edge research hotspot in the field of natural bioactive factors. This review systematically summarizes the natural resource distribution and biological signaling pathways of PA_2_, as well as the in vivo metabolic profiles of Sec. It further elaborates on the core physiological and pathological processes co-regulated by PA_2_ and Sec, including oxidative stress homeostasis, inflammatory immune responses, and metabolic reprogramming. Malignant tumors, severe diseases triggered by disrupted organismal homeostasis, serve as the primary research model for exploring the synergistic regulatory effects of PA_2_ combined with Sec. Accumulating evidence has validated the independent anti-tumor efficacy of PA_2_ and Sec in respective research domains; nevertheless, studies investigating their synergistic bioactivities remain scarce. Starting from the natural resource distribution of PA_2_ and the respective core biological functions of the two agents, this work systematically dissects their individual roles in modulating oxidative stress, inflammatory responses, tumor malignant proliferation and apoptosis, and proposes putative synergistic regulatory mechanisms with hypothetical mechanistic validation. The findings are expected to provide theoretical references and innovative research strategies for developing combinatorial interventions of natural polyphenols and selenocysteine against tumors and related disorders, as well as for constructing antioxidant damage defense systems.

## 1. Introduction

Procyanidins are a class of polyphenolic compounds widely existing in the plant kingdom, composed of flavan-3-ol units. They have attracted much attention due to their significant biological activities such as antioxidant, anti-inflammatory and cardiovascular protective effects. Among them, type A procyanidins, especially procyanidin A_2_ (PA_2_), exhibit stronger biological activity and research value due to their unique molecular structure, which has an additional C_2_-O-C_7_ ether bond connection besides the common C_4_-C_6_ bond. PA_2_ is widely present in a variety of plant resources, such as cranberry (*Vaccinium macrocarpon* Ait.), litchi peel (*Litchi chinensis*), peanut skin *(Arachis hypogaea*) and grape seed (*Vitis vinifera*). These plants have become important sources for the development of health products due to their rich PA_2_ content [1,2,3,4]. PA_2_ exerts its biological functions through a variety of mechanisms, including directly scavenging free radicals, regulating the Nrf_2_ signaling pathway to enhance the expression of intracellular antioxidant enzymes, inhibiting inflammatory responses and abnormal proliferation and migration of vascular smooth muscle cells, thus showing great potential in preventing and improving various chronic diseases [5,6]. At the same time, selenium (Se), as an essential trace element in humans, plays an indispensable role in maintaining body health through its unique organic form, selenocysteine. Selenocysteine is the active center of a variety of selenoproteins, which play a core role in maintaining cellular redox homeostasis, regulating immune function and repairing DNA damage [7]. Selenocysteine effectively scavenges reactive oxygen species through enzymatic reactions, protects cells from oxidative stress damage, and synergizes with other antioxidants such as vitamin E to further enhance antioxidant defense capacity. In addition, selenium biofortification technology also provides an effective way to improve the nutritional value of agricultural products. Selenium treatment can promote the accumulation of anthocyanins in wheat and affect the polyphenols and antioxidant activity in broccoli [8]. Oxidative stress and chronic inflammation are the common pathological basis of many modern chronic diseases, including cardiovascular diseases, neurodegenerative diseases, metabolic syndrome and certain cancers. In view of the unique antioxidant and anti-inflammatory mechanisms of PA_2_ and selenocysteine, it is of great theoretical and practical significance to explore their synergistic effect and the potential of their combined application in health and diseases. PA_2_ regulates the production of antioxidant enzymes by activating the endogenous antioxidant defense system, while selenocysteine, as the active site of key selenoproteins, directly performs enzymatic reactions to scavenge free radicals [5,7]. This complementary mode of regulation and execution indicates that the combined application of the two may produce a more significant synergistic effect than single intervention, providing new ideas for the development of new functional foods, dietary supplements and adjuvant treatment strategies. This review aims to deeply explore the synergistic effects of PA_2_ and selenocysteine, and the possibility of their combined application in antioxidant, anti-inflammatory and cardiovascular protection. Through literature search, there is no combination of selenocysteine and procyanidin A_2_ so far. By integrating the existing research results, this paper will elaborate on the molecular interaction mechanisms of these two bioactive substances, evaluate their combined intervention potential in coping with oxidative stress-related diseases, and provide clues for further understanding the possibility of realizing their mechanisms.

## 2. Plants Containing Procyanidin A_2_

**Figure 1:**
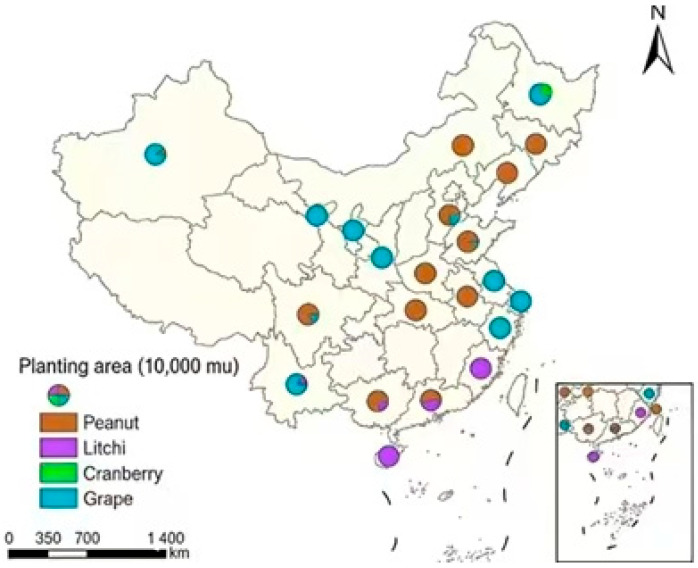
It generally reflects the climatic adaptability and planting area differences of different crops. Peanut (brown): widely distributed in North China, Central China, South China and other regions, with the widest planting range. Grape (blue): mainly distributed in Northwest China, North China and the eastern coastal areas. Cranberry (green): only a small amount distributed in Northeast China. Litchi (purple): concentrated in South China and Hainan, with obvious regional characteristics.

Procyanidin A_2_ is a type A procyanidin widely present in a variety of plants, especially rich in cranberries, litchi peels, peanut skins and grape seeds. This polyphenolic compound has attracted much attention due to its unique structure and significant biological activity. As shown in Figure 1.

### 2.1. Cranberry (Vaccinium macrocarpon)

**Figure 2:**
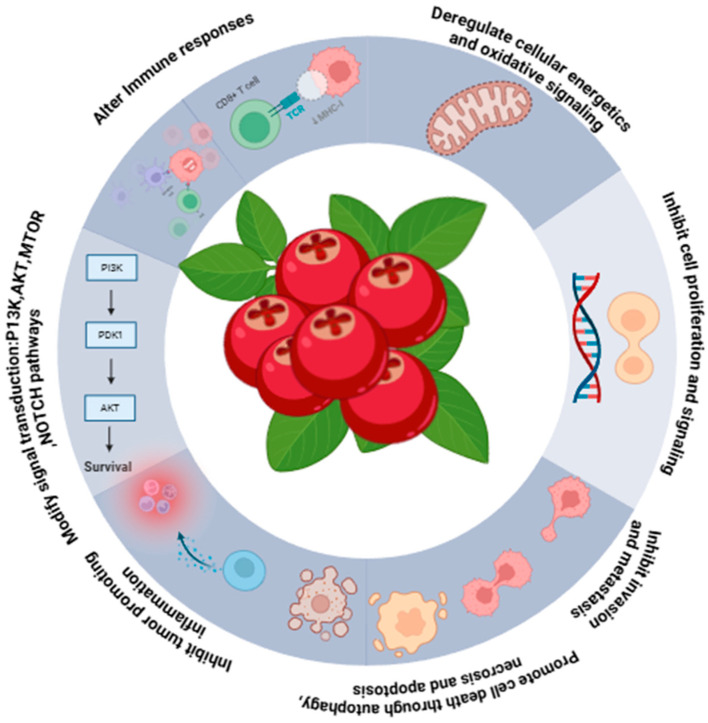
This figure shows the anti-tumor mechanism of cranberry: it can regulate PI3K/AKT/MTOR, NOTCH and other signaling pathways, alter immune responses, regulate cellular energy metabolism and oxidative signaling, inhibit tumor cell proliferation, invasion and metastasis and inflammatory responses, and promote tumor cell death through autophagy, necrosis and apoptosis pathways.

Cranberry is one of the most famous natural dietary sources of PA_2_, especially known for its high content of type A procyanidins [9,10,11]. Type A procyanidins in cranberries are widely regarded as the main active components for their function of preventing urinary tract infection (UTI), which is related to their ability to inhibit bacterial adhesion to uroepithelial cells [11]. Studies have shown that the content and molecular weight distribution of procyanidins in cranberries are key indicators to evaluate their quality and biological activity [12]. Cranberries are not only rich in PA_2_, but also contain a variety of phenolic acids, anthocyanins and flavonoids. These bioactive components jointly endow cranberries with strong antioxidant capacity and a variety of health benefits. Cranberries and their extracts, especially the parts rich in type A procyanidins, have shown significant anti-cancer potential in many studies [9,10]. Its anti-cancer effect is mainly attributed to its strong antioxidant and anti-inflammatory properties [10,13]. Cranberry procyanidins can inhibit cancer cell proliferation, induce cell apoptosis, inhibit angiogenesis and tumor metastasis.As shown in Figure 2. In addition, due to the unique role of cranberries and their active components in the genitourinary system, they also show good prospects in the prevention and adjuvant treatment of genitourinary system tumors such as bladder cancer and prostate cancer. The metabolism of cranberry procyanidins by intestinal microorganisms may also affect their anti-cancer activity. Healthy intestinal microorganisms can produce higher concentrations of phenolic acid metabolites, while the metabolic capacity of intestinal flora in patients with ulcerative colitis for cranberry procyanidin dimers will change [14]. Such differences in the intestinal microbial metabolism may affect the bioavailability and anti-cancer effects of PA2 and its metabolites in gastrointestinal cancers such as colon cancer.

### 2.2. Litchi Peel (Litchi chinensis)

Litchi peel is also an important source of PA_2_ [1]. Studies have found that the content and composition of type A procyanidins in litchi peel change significantly at different stages of litchi fruit development. Especially in the early stage of fruit development, the total phenol content of litchi peel is high, and it shows good antioxidant activity. 11 kinds of procyanidins have been identified in litchi fruit, including procyanidin A_2_, A_4_ and a variety of dimers, trimers and tetramers of type A procyanidins. Although the content of type A procyanidins decreases with fruit ripening, type A procyanidins can exist stably in the young fruit stage of litchi. Litchi peel extract, especially its rich type A procyanidins, has been proved to have the ability to inhibit proliferation and induce cancer cell apoptosis. Polyphenolic compounds in litchi peel can inhibit the growth of a variety of human cancer cell lines by activating the endogenous apoptotic pathway. Although there are relatively few specific anti-cancer studies on PA_2_ in litchi peel, as an important type A procyanidin, PA_2_ is expected to play a synergistic effect in cancer prevention and treatment through its antioxidant stress and anti-inflammatory effects.

### 2.3. Peanut Skin (Arachis hypogaea)

Peanut skin, as the red seed coat outside peanut kernel, is usually regarded as agricultural waste, but it is rich in type A procyanidins, especially PA_2_ [13,15]. A study compared procyanidins in peanut skins and grape seeds, and found that procyanidins in peanut skins are mainly type A, and the polymerization degree of type A procyanidins in peanut skins is usually lower than that in cranberries. The content of type A procyanidin dimers in peanut skins is higher than that in cranberries, while the content of trimers and tetramers is higher in cranberries [13]. PA_2_ in peanut skin is one of its signature components, especially prominent in red peanut varieties. In addition, procyanidins in peanut skin extract show significant antioxidant capacity and anti-cancer potential. Peanut skin procyanidin extract has obvious in vitro inhibitory effects on colon cancer, breast cancer and liver cancer cells. Its anti-cancer mechanism may involve regulating cyclins, inhibiting NF-κB pathway activation, and reducing DNA damage induced by oxidative stress.

### 2.4. Grape Seed (Vitis vinifera)

Grape seed is another important source of procyanidins, but different from cranberries and peanut skins, the content of PA_2_ in grape seeds is relatively low, mainly type B procyanidins, and PA_2_ usually coexists with B_1_/B_2_ as a minor component [15,16]. Grape seed extract (GSE) is rich in procyanidin monomers and oligomers, which are the main bioactive components [16,17]. Although the content of PA_2_ is not high, grape seed procyanidins have attracted wide attention due to their diverse health benefits, such as antioxidant, anti-cancer, anti-diabetic, neuroprotective and antibacterial effects [17,18,19]. Grape seed extract (GSE), rich in procyanidins, has been widely studied for its anti-cancer activity [18,19]. GSE has been proved to have inhibitory effects on a variety of cancer types, such as breast cancer, prostate cancer, colon cancer, lung cancer and skin cancer [16]. Its anti-cancer mechanisms include inducing cancer cell apoptosis, inhibiting cancer cell proliferation, blocking cell cycle, inhibiting angiogenesis and metastasis, and enhancing the sensitivity of chemotherapy drugs [17]. PA_2_ may synergize with other procyanidins to exert anti-cancer effects together. In particular, low-polymerization procyanidins in grape seeds, including possible PA_2_ dimers and trimers, have been found to have significant cytoprotective effects and can protect PC-12 neuroblastoma cells from oxidative stress damage induced by H_2_O_2_ [18]. This indicates that PA_2_ and other oligomeric procyanidins in grape seeds have potential application value in cancer prevention and adjuvant treatment, especially in neuroprotection.

## 3. Pharmacological Effects of Procyanidin A_2_

Procyanidin A_2_ (PA_2_), a type A procyanidin, is widely present in plants, especially in high contents in cranberries, peanut skins, litchi peels and grape seeds [20,21]. Itsunique structure endows it with diverse pharmacological activities, mainly including antioxidant, anti-inflammatory, cardiovascular protection, neuroprotection and potential anti-tumor and antiviral effects [22,23,24,25].

### 3.1. Anti-Cancer Activity

**Figure 3:**
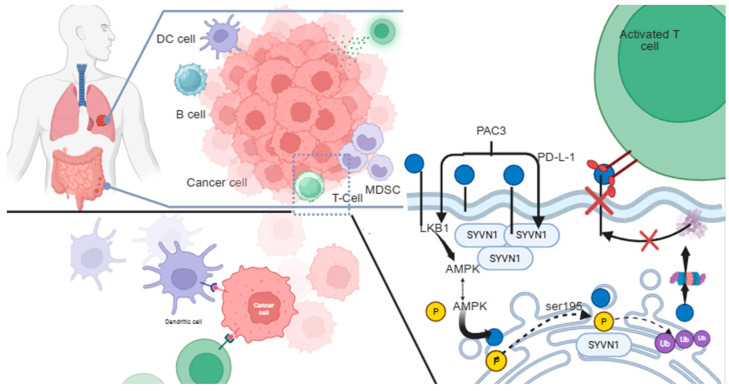
This figure shows the anti-tumor immune regulation mechanism of PA_2_: PA_2_ promotes SYVN1 phosphorylation and membrane translocation by activating the LKB1-AMPK pathway, thereby mediating PD-L1 ubiquitination and degradation, and relieving the inhibition of activated T cells. At the same time, it can reshape the tumor microenvironment, enhance the antigen presentation and killing functions of immune cells such as dendritic cells, and ultimately achieve anti-tumor effects.

As an important class of plant compounds, procyanidins have been widely studied as potential chemopreventive and chemotherapeutic agents for fighting a variety of cancers [26]. Its anti-tumor activity is closely related to its significant antioxidant and anti-inflammatory properties, which can synergistically regulate cell signaling pathways and affect the growth, differentiation, apoptosis and metastasis of cancer cells. Specifically for procyanidin A_2_, studies have explored its mechanism of action in tumor cells. For example, some studies have found that autophagy induced by procyanidins can reduce the apoptosis of human gastric cancer cell MGC-803, suggesting that it may have a complex regulatory effect in tumor cells [27,28]. Inhibit cancer cell proliferation by regulating the cell cycle process and preventing the unlimited proliferation of cancer cells. Induce cancer cell apoptosis and promote the programmed death of cancer cells by activating endogenous or exogenous apoptotic pathways. Inhibit angiogenesis, as tumor growth and metastasis require the supply of new blood vessels, procyanidins can inhibit tumors by inhibiting blood vessel formation as shown in Figure 3. Owing to their multi-modal pharmacological activity and the inherent safety profile of natural polyphenols, procyanidins hold significant promise as agents for both cancer chemoprevention and adjuvant treatment.

### 3.2. Antioxidant Activity

**Figure 4:**
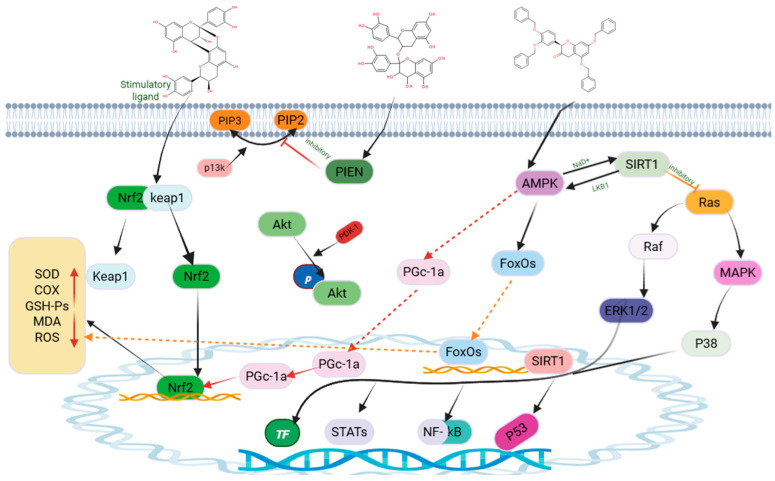
The mechanism of procyanidins (PACs) exerting antioxidant and cytoprotective effects by regulating multiple signaling pathways: Nrf_2_ pathway: activate Nrf_2_ and promote its nuclear translocation, up-regulate the expression of antioxidant enzymes such as SOD and GSH-Px, and reduce the level of oxidative stress such as ROS and MDA. PI3K/Akt pathway: inhibit PI3K/Akt activation and reduce proliferative signals. AMPK/PGC-1α pathway: activate AMPK, up-regulate PGC-1α, and synergize with Nrf_2_ to enhance antioxidant and mitochondrial functions. MAPK/inflammatory pathway: inhibit MAPK pathways such as ERK1/2 and p38, and inflammatory transcription factors such as NF-κB and STATs, to reduce inflammatory responses.

Proanthocyanidin A_2_ (PA_2_) exhibits potent antioxidant activity. In vitro assays have demonstrated that PA_2_ effectively scavenges free radicals and alleviates tert-butyl hydroperoxide (t-BHP)-triggered oxidative injury in HepG_2_ hepatocytes [29]. This cytoprotective effect is primarily attributed to the activation of the nuclear factor erythroid 2-related factor 2 (Nrf2) signaling pathway. As the master transcription factor governing cellular antioxidant defense against oxidative stress, Nrf2 strengthens cellular resistance by upregulating the transcription of antioxidant enzyme genes. In lipopolysaccharide (LPS)-stimulated RAW264.7 macrophage models, PA_2_ also activates the Nrf2 cascade and suppresses excessive intracellular accumulation of reactive oxygen species (ROS) As shown in Figure 4. ROS are physiological byproducts of cellular metabolism. While high ROS concentrations trigger oxidative stress and cellular damage, low levels of ROS participate in the regulation of physiological signaling. Cells rely on an integrated antioxidant network to sustain redox homeostasis. Nrf2 pathway activation by PA_2_ generates synergistic effects with the selenoprotein-based antioxidant system mediated by selenocysteine (Sec). Co-administration of PA_2_ and Sec jointly eliminates surplus ROS, attenuates apoptosis and inflammation induced by redox imbalance, and confers potential protective efficacy in damaged normal tissue repair. Nevertheless, this robust antioxidant capacity is dual-edged, which raises the well-documented antioxidant paradox in oncology research. Most current anti-tumor therapeutic regimens eliminate malignant cells by elevating intracellular ROS levels to provoke oxidative damage or ferroptosis [22]. Sustained reinforcement of antioxidant capacity via combined PA_2_ and Sec treatment may neutralize toxic ROS within the tumor microenvironment, thereby restraining ferroptosis and oxidative damage-mediated tumor cell death. Persistently activated Nrf2 further reinforces the antioxidant shield of tumor cells, blunting the responsiveness to ROS-dependent therapies and even driving the emergence of drug resistance.

### 3.3. Anti-Inflammatory Activity

**Figure 5:**
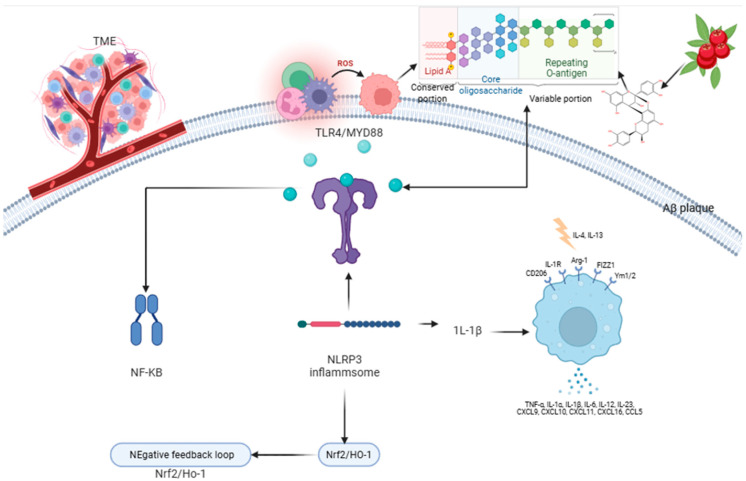
This figure shows the regulatory mechanism of procyanidins in tumor microenvironment (TME) and neuroinflammation: scavenge ROS, inhibit the activation of TLR4/MYD88 pathway and NLRP3 inflammasome, and reduce the release of pro-inflammatory factors such as IL-1β. Activate the Nrf_2_/HO-1 pathway to form a negative feedback, alleviate inflammation and reduce Aβ plaque-related nerve damage. Inhibit NF-κB and other pathways, regulate immune cell functions, and ultimately exert anti-inflammatory, neuroprotective and anti-tumor potential.

The anti-inflammatory effect of PA2 has been confirmed by many studies [22,23,25]. In LPS-stimulated RAW264.7 cells, PA2 can dose-dependently inhibit the production of inflammatory mediators, such as nitric oxide, prostaglandin E_2_, and the expression of pro-inflammatory cytokines interleukin-6 and tumor necrosis factor-α [22]. Its anti-inflammatory mechanism mainly involves the regulation of multiple signaling pathways: NF-κB pathway inhibition: PA_2_ can inhibit LPS-induced NF-κB phosphorylation and nuclear translocation, thereby reducing the expression of NF-κB target genes, which encode a variety of pro-inflammatory factors. MAPK pathway regulation: PA_2_ exerts anti-inflammatory effects by inhibiting the phosphorylation of MAPK family members such as p38, ERK and JNK. COX-2 inhibition: PA_2_ can inhibit the expression of cyclooxygenase-2 (COX-2), a key enzyme for the production of a large number of pro-inflammatory prostaglandins in the inflammatory response [22], as shown in Figure 5. Relevant studies also show that procyanidins can inhibit the synthesis of PGE_2_ in LPS-induced RAW264.7 cells, which may be related to COX-2 enzyme activity. Inflammation is a complex process involving the production of eicosanoids, metabolites of arachidonic acid. These molecules can both initiate inflammation and promote inflammation resolution [29]. Non-steroidal anti-inflammatory drugs work by inhibiting COX enzymes, but may be accompanied by gastrointestinal side effects.

### 3.4. Cardiovascular Protective Effect

Procyanidin A_2_ and its parent compound procyanidins have multiple protective effects on the cardiovascular system. Improvement of endothelial function: procyanidins can inhibit the proliferation and migration of vascular smooth muscle cells, which is crucial for the prevention and treatment of vascular diseases such as atherosclerosis [23]. PA_2_ can inhibit PDGF-BB-induced VSMC proliferation and migration, and its mechanism may be related to the inhibition of KDR and Jak-2/STAT-3/cPLA2 signaling pathways. Procyanidins can inhibit thrombosis by affecting platelet activity. Grape seed procyanidins have been proved to reduce doxorubicin-induced cardiotoxicity [30,31]. Doxorubicin is an important anti-cancer drug, but its clinical application is limited by cardiotoxicity, which is mainly attributed to the accumulation of reactive oxygen species [30]. The antioxidant properties of procyanidins help to fight this toxicity [31].

### 3.5. Neuroprotective Potential

Procyanidin compounds have neuroprotective effects. Grape seed procyanidins have a protective effect on oxidative stress of PC-12 neuroblastoma cells induced by H_2_O_2_, indicating their neuroprotective potential [32]. Although there are relatively few studies on the direct neuroprotection of PA_2_ at present, its derivative procyanidin B_2_ has been proved to protect neurons from oxidative stress, nitrosative stress and excitotoxicity [33]. Metabolites of procyanidins may also cross the blood-brain barrier to exert neuroprotective effects [24]. In addition, A_2_ receptor antagonists show potential in the treatment of Parkinson’s disease because they can improve motor symptoms [34].

## 4. Selenium

Selenium (Se) is an essential trace element crucial to human health, exerting its biological functions mainly by being integrated into selenoproteins. Selenium has various chemical forms, roughly divided into inorganic selenium and organic selenium. Among them, selenomethionine and selenocysteine are the two main forms of organic selenium, which play different roles in organisms and show significant differences in physiological functions, metabolic regulation and bioavailability [35,36]. As shown in Table 1.

### 4.1. Selenium Element

Selenium is a trace element with multifunctional redox activity, and its biological effects depend on its chemical form and dosage [37,38,39]. The content of selenium in soil on the earth is different, leading to differences in dietary selenium intake worldwide [40]. Appropriate selenium intake is crucial for maintaining the body’s antioxidant defense, thyroid hormone metabolism, immune function and reproductive health [40,41,42]. Selenium deficiency is associated with a variety of diseases, such as Keshan disease and Kashin-Beck disease, while excessive intake may cause toxic reactions.

### 4.2. Selenomethionine

Selenomethionine is an organic selenium form and the main storage form of selenium in plants [36]. In organisms, SeMet can non-specifically replace methionine in ordinary proteins and incorporate into protein chains [43]. This incorporation process does not depend on a specific genetic code recoding mechanism, but shares the metabolic pathway with methionine. Because SeMet can be incorporated into any protein as an analog of methionine, it has high bioavailability in organisms, but its functional activity regulation in cells is poor. The accumulation of SeMet in proteins makes it the main storage form of selenium. When needed, it can be degraded to release selenium for the synthesis of selenoproteins [35]. In X-ray crystallography, labeling proteins by replacing methionine with SeMet can use the anomalous scattering signal of selenium to resolve protein structures, which is crucial for solving the phase problem and determining protein structures.

### 4.3. Selenocysteine (Sec)

Selenocysteine is the 21st naturally encoded amino acid, whose structure is similar to cysteine but with the sulfur atom replaced by a selenium atom [44,45,46]. This tiny change endows Sec with stronger nucleophilicity and unique redox properties than cysteine, making it show higher activity and efficiency in catalytic reactions [44,45,46,47]. The incorporation of Sec is a highly specific and strictly regulated process, which is realized through the genetic recoding mechanism of the UGA stop codon [45,48,49,50]. This process requires a series of complex molecular machinery, including special selenocysteine tRNA, specific elongation factors and selenocysteine insertion sequence on mRNA [45,46,48,49]. The SECIS element is a secondary structure on mRNA, usually located near the UGA codon in bacteria, while in eukaryotes, it is located in the 3′ untranslated region of mRNA, guiding the recoding of UGA codon through long-distance interaction.

### 4.4. Selenoproteins

Selenoproteins are a class of proteins containing selenocysteine, which are the main carriers for selenium to exert biological functions [35,36,37]. The human genome encodes 25 selenoproteins, which play key roles in a variety of physiological processes, including antioxidant defense, thyroid hormone metabolism, immune response, cellular redox homeostasis and neural development [40,44,51,52,53]. Glutathione peroxidase (GPx) and thioredoxin reductase are important antioxidant selenoproteins, which effectively scavenge reactive oxygen species and protect cells from oxidative damage by integrating selenocysteine into their active centers [37,41,54,55,56]. GPX4, as one of them, is a key regulator of ferroptosis [54,56]. Iodothyronine deiodinase is another selenoprotein that plays an indispensable role in the activation and metabolism of thyroid hormones [40].

In summary, selenocysteine plays an irreplaceable role in maintaining body redox balance, regulating immune function, promoting neural development, etc [37,42,53,57]. Selenium promotes neural development in neuronal models by regulating glutathione peroxidase 4 (GPX4) and selenoprotein P. Selenoproteins play a key role in fighting oxidative stress through their contained Sec residues, and oxidative stress is closely related to the occurrence and development of a variety of chronic eye diseases [55]. Although various forms of selenium are beneficial to health, selenocysteine shows more significant scientific rationality and clinical application value in nutritional intervention, precise selenium supplementation and the development of functional foods or drugs due to its unique physiological specificity, precise metabolic regulation mechanism, higher biological potency and better safety.

## 5. Physiological Functions of Selenocysteine

As a unique amino acid, selenocysteine plays a crucial role in orga nisms, and its physiological functions are mainly exerted as the active center of selenium-containing proteins, endowing these proteins with enhanced catalytic and redox properties. This unique property makes it play a core role in maintaining cellular homeostasis, resisting diseases, especially in antioxidant, anti-tumor, anti-inflammatory and many other physiological processes [58,59].

### 5.1. Antioxidant Effect

Maintenance of antioxidant defense and redox homeostasis Selenocysteine is a key component of the cellular antioxidant defense system, especially in the active sites of a variety of selenium-containing antioxidant enzymes. These enzymes work synergistically to effectively scavenge reactive oxygen species, thereby protecting cells from oxidative damage [58]. Glutathione peroxidase (GPx) family: GPx is an important selenium-containing enzyme whose catalytic activity mainly depends on the presence of selenocysteine [58,60]. GPx effectively scavenges ROS by reducing hydroperoxides and lipid peroxides to the corresponding alcohols, thereby protecting biological macromolecules such as cell membranes and DNA from oxidative damage. Glutathione peroxidase 4 is a key regulator of ferroptosis [60,61]. Ferroptosis is a form of programmed cell death closely related to lipid peroxidation. Abnormal GPX4 function is closely related to a variety of neurodegenerative diseases, cancers and cardiovascular diseases [60]. Dietary selenium intake directly affects the activity level of GPx. Selenium deficiency will lead to decreased GPx activity, thereby weakening the antioxidant capacity of cells [62]. Thioredoxin reductase (TrxR) family: TrxR is also a class of enzymes containing selenocysteine [63,64]. They play a key role in regulating cellular redox state, DNA synthesis, cell proliferation, cell apoptosis and transcription factor activity by maintaining the reduced state of the thioredoxin system [63]. Selenocysteine endows TrxR with resistance to oxidative inactivation, which is crucial for the maintenance of enzyme activity, especially compared with cysteine homologs, the presence of selenocysteine makes TrxR more resistant to inactivation by oxidants such as H_2_O_2_ [64]. The activity of TrxR is also affected by dietary selenium levels. The high efficiency of selenocysteine comes from its unique chemical structure. The low pKa value of its thiol group makes it almost completely in an active state at physiological pH, thus showing higher nucleophilicity and catalytic efficiency than cysteine. Studies have shown that at any given time, more than 99% of Sec residues are in a “ready” state, while only about 11% of cysteine residues are [65]. This characteristic makes Sec-containing enzymes have higher efficiency in antioxidant aspects and can catalyze redox reactions at a faster rate.

### 5.2. Anti-Tumor Activity

**Figure 6:**
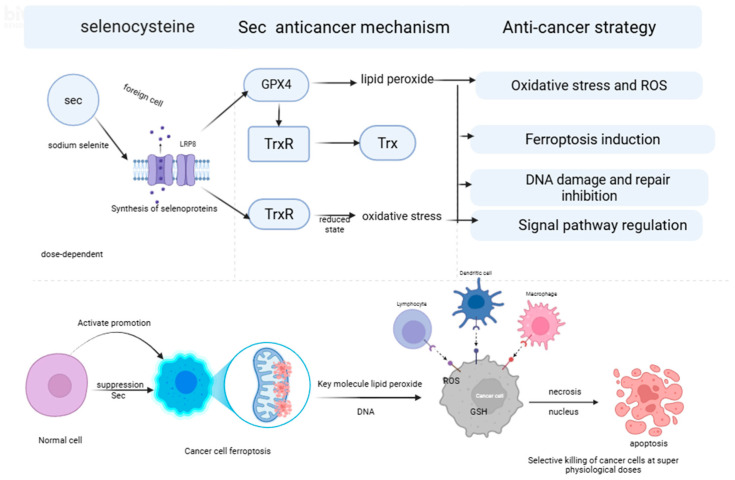
Anti-tumor mechanisms and strategies of selenocysteine (Sec): Molecular level: Sec participates in the synthesis of selenoproteins such as GPX4 and TrxR, and regulates lipid peroxidation and oxidative stress. Cellular level: Supraphysiological dose of Sec selectively induces ferroptosis in cancer cells and inhibits their proliferation with little effect on normal cells. Achieve anti-cancer effects through oxidative stress regulation, ferroptosis induction, DNA damage repair inhibition and signal pathway regulation.

Selenium and selenoproteins play complex and multifaceted roles in tumorigenesis and cancer progression, and their effects may depend on selenium concentration, cell type and tumor specificity. Inhibition of tumor development: selenoproteins may inhibit cancer development through their strong antioxidant activity [58,66]. Reduce DNA damage caused by oxidative stress through antioxidant enzymes such as glutathione peroxidase, thereby reducing the risk of mutation. Selenocysteine is also involved in regulating the cell cycle, inhibiting cell proliferation, and inducing tumor cell apoptosis, thus exerting anti-cancer effects. Studies have shown that selenium intake is associated with a reduced risk of a variety of cancers [58]. Mitochondrial function regulation: some studies have also revealed that selenium-containing compounds exert anti-tumor effects by affecting mitochondrial function. Bavachol, a phytoestrogen, was found to inhibit the proliferation of human choriocarcinoma cells by targeting the electron transport chain complex and inducing mitochondrial dysfunction, as shown in Figure 6. This suggests the potential role of selenocysteine in regulating mitochondrial function to fight cancer, but it should be noted that selenium has a “double-edged sword effect” [66]. Although selenium has the potential to prevent cancer at lower concentrations, it or selenoproteins may also promote the survival and growth of tumor cells at high concentrations or in specific cancer backgrounds, which is usually related to the enhanced adaptability of tumor cells to oxidative stress.

### 5.3. Anti-Inflammatory Effect

Selenium and selenoproteins also play an important role in regulating immune responses and inflammatory processes. Selenoproteins are essential for the normal operation of the immune system. Some selenoproteins are involved in endoplasmic reticulum stress response, calcium homeostasis and immune regulation [60]. Mitochondria are also crucial for immune cell function. Mitochondrial complex III is found to be crucial for the inhibitory function of regulatory T cells, thus playing a role in immune tolerance [67]. Through its role in selenium-containing proteins, selenium can affect the activity and signal pathways of immune cells, thus possibly regulating inflammatory responses.

### 5.4. Other Physiological Functions

Selenocysteine is also involved in many other key physiological processes and is crucial for maintaining the overall health of the body. Regulation of thyroid hormone metabolism: selenocysteine is a component of the iodothyronine deiodinase (DIO) family. DIO enzymes play a key role in the activation and inactivation of thyroid hormones, responsible for converting thyroxine (T4) into biologically active triiodothyronine (T3), or converting T4 and T3 into inactive metabolites. This local regulation is crucial for maintaining systemic metabolic homeostasis, especially for complex processes such as fetal brain development and adaptive responses to diseases [59]. Selenium deficiency will lead to impaired DIO enzyme function, thereby affecting thyroid function. Selenium transport and storage: selenoprotein P is the main selenium transport protein in plasma [68,69]. Human SELENOP contains 10 selenocysteine residues, one of which is located at the N-terminus with redox function, and the remaining 9 are located at the C-terminus as selenium transport and storage sites. It is not only responsible for transporting selenium from the liver to other tissues, but also has antioxidant functions [68]. Its biosynthesis is particularly sensitive to UGA codons and is susceptible to ribosomal function interfering agents [69]. Reproductive system function: selenium-containing proteins containing selenocysteine are involved in maintaining the normal function of the male reproductive system in mammals [70]. Selenium deficiency is associated with male infertility, prostate cancer, testicular cancer and other diseases. Bone development: selenium is involved in the formation of selenoproteins. Studies have shown that selenium deficiency will lead to abnormal selenoprotein biosynthesis, which may lead to brain, bone development and chondrocyte differentiation disorders [71]. Neuroprotection: selenium is regarded as an important trace element crucial for nervous system health. As a component of selenium-containing proteins, selenocysteine helps to protect neurons from damage and prevent the occurrence and development of neurodegenerative diseases through antioxidant effects and maintaining cellular homeostasis [72].

## 6. Prospect of the Combination of Procyanidin A_2_ and Selenocysteine

The combined application of procyanidin A_2_ and selenocysteine shows significant synergistic potential in anti-tumor research, and its core mechanism lies in the complementary effects of the two in redox homeostasis, ferroptosis regulation, tumor cell proliferation and metastasis inhibition, and tumor microenvironment regulation [73,74].

### 6.1. Synergistically Enhance Antioxidant Defense

Procyanidin A2 enhances the cellular antioxidant defense capacity by activating the nuclear factor E2-related factor 2 (Nrf_2_) pathway and up-regulating the expression of endogenous antioxidant enzymes [74,75]. Nrf_2_ is a key regulatory factor for cells to respond to oxidative stress, and its activation can promote the transcription of more than 250 antioxidant genes related to redox balance, mitochondrial biogenesis, metabolism, detoxification and cell protection. For example, the latest research points out that Nrf_2_ regulates more than 250 genes related to redox balance, mitochondrial biogenesis, metabolism, detoxification and cell protection [76]. PA2 can promote Nrf2 nuclear translocation by modifying the cysteine residues of Keap1 protein or interfering with the binding of Keap1 and Nrf2, thereby activating the expression of genes driven by antioxidant response elements [74]. Selenocysteine, as the 21st gene-encoded amino acid, is a component of the active sites of a variety of selenoproteins [73,77]. In particular, glutathione peroxidase 4 (GPX4) is a selenium-containing cysteine enzyme, a key defense enzyme for cells to resist ferroptosis, which can effectively scavenge lipid peroxides in cell membranes and prevent cell damage caused by lipid peroxidation [73,77,78,79]. Selenocysteine is integrated into these enzymes through a unique SECIS-dependent translation mechanism to ensure the precise presence of selenium at the catalytic active site. Therefore, PA_2_ activates the Nrf_2_ pathway to enhance the overall capacity of the endogenous antioxidant system at the transcriptional level, while selenocysteine provides essential catalytic factors for core antioxidant enzymes such as GPx to achieve efficient scavenging of reactive oxygen species (ROS) [73,74]. Nevertheless, numerous published studies have indicated that excessive antioxidant capacity confers protective effects on tumor cells. However, intracellular ROS detection assays conducted in our laboratory have verified that selenocysteine (Sec) combined with proanthocyanidin A_2_ (PA_2_) can markedly inhibit the proliferation of tumor cells at specific treatment concentrations. Our research group has obtained encouraging preliminary experimental findings concerning their combined application. The co-treatment of Sec and PA_2_ is capable of alleviating oxidative stress within the tumor microenvironment more efficiently, shielding normal cells from injuries induced by chemotherapy or radiotherapy, and potentially restraining aberrant proliferation of tumor cells [80,81].

### 6.2. Strengthen Ferroptosis Induction

**Figure 7:**
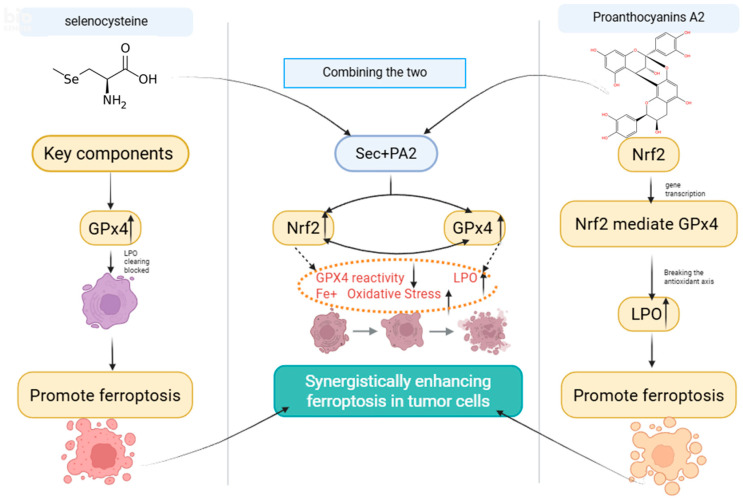
The mechanism of selenocysteine (Sec) and procyanidin A_2_ (PA_2_) synergistically inducing ferroptosis in tumor cells: Sec: down-regulates GPX4, blocks lipid peroxide (LPO) scavenging, and directly promotes ferroptosis. PA_2_: activates Nrf_2_ and mediates GPX4 expression, breaks the antioxidant axis, and accumulates LPO to promote ferroptosis. Combined effect (Sec + PA_2_): through the interactive regulation of Nrf_2_ and GPX4, aggravate oxidative stress and LPO accumulation, synergistically enhance ferroptosis in tumor cells, and achieve efficient anti-tumor effects.

Ferroptosis is an iron-dependent form of programmed cell death driven by the accumulation of lipid peroxides [81]. The activity of GPX4 is a key factor in inhibiting ferroptosis, because it can reduce lipid hydroperoxides to non-toxic lipid alcohols, thus blocking the chain reaction of lipid peroxidation [73,82]. Selenocysteine is a key component for GPX4 to exert its functions. Selenium supplementation can protect cells from ferroptosis by enhancing the abundance of GPX4 [77,83]. Studies have shown that patients with significantly reduced GPX4 enzyme activity caused by gene mutations are highly sensitive to ferroptosis [82]. If tumor cells are sensitive to ferroptosis, the regulatory effect of PA_2_ on the Nrf2 pathway can indirectly affect the expression of GPX4, because Nrf_2_ can up-regulate the transcription of a variety of antioxidant genes including GPX4 [84,85]. By precisely regulating the level of selenocysteine and combining with the regulation of Nrf_2_ by PA_2_, the activity of GPX4 can be synergistically regulated to induce ferroptosis in tumor cells more effectively [81,86]. The combined use of targeted GPX4 inhibitors and CDK_4_ inhibitors can induce phospholipid remodeling through cell cycle arrest, thereby making cancer cells more sensitive to ferroptosis triggered by GPX4 inhibitors [86] as shown in Figure 7. On the other hand, the activation of the Nrf_2_ pathway has a cytoprotective effect on normal cells [74,78]. This protective mechanism needs to be fully considered in tumor treatment to reduce side effects. In a cerebral ischemia model, USP15 regulates ferroptosis and cognitive dysfunction through the Nrf2/GPX4 axis [85]. However, in some tumors with excessive Nrf_2_ activation, Nrf_2_ may also become a mechanism of tumor drug resistance [87]. At this time, it may be necessary to adjust the dose of PA_2_ or combine with Nrf2 inhibitors to avoid the survival advantage of tumor cells.

### 6.3. Multi-Target Inhibition of Tumor Cell Proliferation and Metastasis

The combinatorial therapeutic strategy of proanthocyanidin A2 (PA2) and selenocysteine (Sec) holds promising therapeutic value for pancreatic ductal adenocarcinoma (PDAC). This hypothesis is grounded in the strong molecular complementarity between the two agents and their precise targeting of the unique pathological hallmarks of PDAC. PDAC is a lethal malignancy characterized by persistent activation of KRAS-driven signaling cascades; its survival and metastatic progression are heavily reliant on aberrant activation of the ERK1/2 and PI3K/Akt pathways, alongside a chronic inflammatory microenvironment mediated by NF-κB [88]. As a dimeric proanthocyanidin, PA2 has been validated to directly suppress the phosphorylation of ERK1/2 and Akt, thereby ablating downstream proliferative signaling [89]. By downregulating cyclin D1 and CDK4 while upregulating the cell cycle suppressors p21 and p27, PA2 efficiently arrests tumor cells at the G1 phase and restrains their uncontrolled proliferation [90]. Furthermore, PA2 markedly represses the secretion of matrix metalloproteinases MMP-2 and MMP-9, two pivotal enzymes that enable tumor cells to degrade the basement membrane and facilitate invasion and metastasis [91]. Meanwhile, as the core building block of selenoproteins, selenocysteine plays an indispensable role in sustaining genomic stability and governing redox homeostasis. Selenoproteins such as thioredoxin reductase (TrxR) not only participate in reactive oxygen species (ROS) scavenging but also serve critical functions in DNA repair machinery [92]. In PDAC, malignant cells frequently hijack the Nrf2 pathway to boost antioxidant defense and counteract oxidative damage triggered by chemotherapy. Appropriate selenium intervention disrupts this cytoprotective adaptation via modulation of the Nrf2/HO-1 axis [93]. More importantly, selenium compounds have been proven to inhibit NF-κB-mediated transcription of pro-metastatic genes and disrupt integrin signaling, ultimately impairing the migratory capacity of tumor cells [94]. Studies on hepatocellular carcinoma have demonstrated that Sec precursors or selenium agents potentiate the anti-tumor efficacy of chemotherapeutics including cisplatin, with underlying mechanisms encompassing suppression of DNA repair enzyme activity and induction of mitochondria-dependent apoptosis [95]. Integrating the direct anti-proliferative and anti-migratory functions of PA2 with Sec’s regulatory roles in genomic stability and oxidative stress sensitization yields a multi-pronged therapeutic strategy against PDAC. PA2 blocks upstream driver signals (ERK/Akt) and neutralizes invasion mediators (MMPs), whereas Sec disrupts intracellular redox balance and DNA repair capacity to reverse chemoresistance in tumor cells [96]. This synergistic effect combining “signaling blockade and metabolic vulnerability induction” is particularly advantageous for overcoming the clinical hurdles of dense stromal barriers and profound drug resistance characteristic of PDAC [97]. Given that most PDAC patients are diagnosed at advanced stages and exhibit limited responsiveness to conventional chemotherapy, the combined PA2-Sec regimen is expected to suppress tumor progression through multi-targeted mechanisms, including triggering tumor cell apoptosis, attenuating distant metastasis, and sensitizing standard chemotherapeutic protocols [96].

### 6.4. Regulate Inflammation in the Tumor Microenvironment

**Figure 8:**
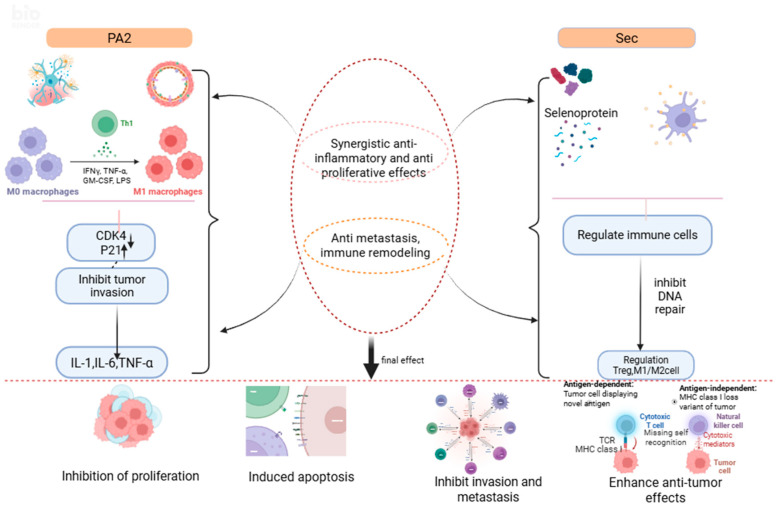
This figure shows the synergistic anti-tumor effect of procyanidin A_2_ (PA_2_) and selenocysteine (Sec): PA_2_: regulates macrophage polarization, down-regulates CDK4, up-regulates P21, inhibits tumor invasion, and reduces pro-inflammatory factors such as IL-1β, IL-6 and TNF-α at the same time, exerting anti-inflammatory and anti-proliferative effects. Sec: participates in selenoprotein synthesis, regulates immune cell function, inhibits DNA repair, regulates Treg/M1/M2 cell subsets, and enhances anti-tumor immunity. Combined effect: through the synergistic effects of anti-inflammatory, anti-proliferative, anti-metastatic and immune remodeling, the two ultimately achieve the effects of inhibiting tumor growth, inducing apoptosis and strengthening immune killing.

Inflammation is one of the important factors in tumorigenesis and development [81,97]. Sustained inflammatory response in the tumor microenvironment can promote tumor growth, angiogenesis, immune escape and metastasis. Procyanidins have significant anti-inflammatory activity, which can inhibit the TLR4/MyD88/NF-κB signaling pathway and NLRP3 inflammasome activation, thereby reducing the release of pro-inflammatory cytokines such as IL-1β, IL-6 and TNF-α [76,89]. Selenocysteine has a regulatory effect on immune cell function. Selenium can affect the immune system through a variety of pathways, including enhancing the differentiation of T regulatory cells, promoting the polarization of M1 macrophages to M2 macrophages, and enhancing the cytotoxicity of NK cells through TrxR1-dependent redox control [98]. Studies have shown that the combination of selenium and fish oil can enhance anti-cancer efficacy and regulate the tumor microenvironment through multiple signaling pathways [93,94] as shown in Figure 8. At the same time, both can regulate immune suppressive cell populations such as myeloid-derived suppressor cells (MDSCs) and tumor-associated neutrophils (TANs), which play a key role in immune suppression in the tumor microenvironment [98]. Selenium compounds can reduce the accumulation of MDSCs and the expression of arginase-1, while PA2 can weaken neutrophil infiltration. This combined effect can transform the immune-suppressive, pro-tumor microenvironment into one that is conducive to immune surveillance and the efficacy of checkpoint inhibitors [99,100].

### 6.5. Other Diseases

Pathophysiological studies of neurodegenerative diseases have shown that oxidative stress plays a key role in diseases such as Alzheimer’s disease (AD) and Parkinson’s disease (PD) [101,102,103]. Oxidative stress refers to the imbalance between the production of reactive oxygen species (ROS) and the antioxidant defense system, leading to cell damage. This change in redox state is closely related to the process of cellular senescence, and senescent cells release pro-inflammatory factors, which further aggravate neuroinflammation and neurodegeneration [103]. As an essential trace element, selenium is a key component of selenoproteins, which play an important role in maintaining cellular redox homeostasis [104]. Selenium nanoparticles have shown potential in the treatment of AD due to their unique physical and chemical properties and potential therapeutic effects [105]. The combined application of procyanidin A_2_ with neuroprotective effects and selenocysteine may provide a more effective intervention strategy for these neurodegenerative diseases [101]. Procyanidin A_2_ is a polyphenolic compound with multiple biological activities such as scavenging free radicals, anti-inflammatory and regulating Nrf_2_ and other signaling pathways. The Nrf_2_ pathway is a key defense mechanism for the body to respond to oxidative stress, protecting cells by activating the expression of antioxidant enzyme genes [102]. Therefore, combining the enhancement of endogenous antioxidant defense by selenocysteine with the direct free radical scavenging and Nrf_2_ pathway regulation by procyanidin A_2_ is expected to provide more comprehensive neuroprotection. In terms of metabolic diseases, there is a complex and delicate relationship between selenium and diabetes. Both selenium deficiency and excessive supplementation may cause health problems. Studies have shown that selenium supplements may be associated with an increased incidence of type 2 diabetes [103]. However, selenium plays an important role in regulating glucose metabolism and insulin sensitivity, and appropriate selenium intake is crucial for maintaining metabolic health. Obesity is a risk factor for a variety of metabolic disorders, and weight loss and reduced cardiovascular risk in obese patients are closely related to the successful correction of hyperglycemia [106]. In this context, the combined use of selenocysteine and procyanidin A2 may indirectly benefit metabolic health by improving oxidative stress and inflammatory status. Oxidative stress and chronic inflammation are key driving factors for the development of obesity and type 2 diabetes. Selenocysteine can enhance endogenous antioxidant capacity through selenoproteins, while procyanidin A_2_ can exert anti-inflammatory and antioxidant effects [102]. This combined intervention is expected to synergistically alleviate oxidative stress and inflammation related to metabolic disorders, thereby improving blood glucose control and cardiovascular health.

## 7. Conclusions

This review thoroughly explores the great application potential of two highly bioactive natural compounds, proanthocyanidin A2 (PA2) and selenocysteine (Sec), in the field of health products. We analyze the abundant botanical sources, unique structural characteristics, and diverse biological activities of PA2, as well as the vital physiological functions exerted by Sec—the biologically active form of selenium, an essential trace element for humans. Existing evidence reveals that PA2 and Sec each confer prominent health benefits; moreover, their combined administration may exert synergistic effects and possess promising prospects in antioxidation, anti-inflammation, and cardiovascular protection. Despite the broad translational outlook for combined PA2 and Sec therapy, several challenges remain unaddressed. PA2 suffers from low oral bioavailability, while although Sec maintains favorable therapeutic safety profiles, daily intake exceeding 400 μg may trigger toxic side effects. Additionally, the dose–effect relationship governed by the Nrf2 pathway is intricate and likely follows a biphasic dose–response curve. Future research should focus on nanoparticle co-delivery systems to elevate the oral bioavailability of PA2 and precisely regulate Sec dosage. Furthermore, stratifying patients based on tumor Nrf2 activation status and GPX4 expression levels, followed by mechanistic validation in immunocompetent syngeneic animal models, will facilitate further clarification of their synergistic mechanisms and clinical translational capacity. Although the underlying interactive mechanisms of these two agents remain largely theoretical at present, PA2 and Sec, as vital bioactive molecules, hold tremendous application potential for the prevention and adjuvant treatment of disorders linked to oxidative stress and inflammation.

## Figures and Tables

**Table 1:** Selenium Family.

Selenium Family Members	Selenium (Se, Selenium Elemet)	Selenomethionine (SeMet)	Selenoprotein (Sec, the 21st Standard Amino Acid)	Selenoproteins
Nature	Essential trace non-metal element for human body	Selenium-containing essential amino acid	Functional selenium-containing amino acid encoded by unique genetic code	General term for bioactive proteins with Sec residues
Core Chemical Structure	Elemental inorganic selenium:selenous acid, sodium selenite, sodium selenateorganoselenium bound to carbon chains	CH_3_-Se-CH_2_-CH(NH_2_)-COOH	HS-CH_2_-SeH-CH_2_	Sec is incorporated into polypeptide chains and serves as the active center
Existing Forms	Inorganic selenium (soil, dietary supplements);Organoselenium (plants, animals)	Primary selenium storage form in plants (cereals, soybeans)	Building block of all animal and microbial selenoproteins	Intracellular transport proteins in animals; Synthesized by eukaryotes and prokaryotes
Biosynthetic Source	Absorbed from the environment; cannot be synthesized endogenously in humans	Plants synthesize it via substitution of sulfur with inorganic selenium; humans only obtain it from diet	Humans cannot synthesize it de novo; relies on selenium-serine transformation. Selenium insertion requires the SECIS element	Ribosomes recognize SECIS during translation to integrate Sec into peptide chains
Metabolic Regulation	Regulated by systemic homeostasis; excessive intake causes toxic accumulation	Highly bioavailable; deficiency is precisely regulated, while excess tends to accumulate	Tightly controlled post-transcriptionally via the SECIS mechanism to prevent overaccumulationphysiological pH	Strictly regulated at transcriptional and post-translational levels
Safety	Inorganic selenium has higher toxicity, while organic selenium is relatively safer.	Higher bioavailability, but excessive intake can still cause toxicity	Safer, the endogenous synthesis pathway limits its tissue concentration, and its acute toxicity is significantly lower than that of inorganic selenium and excessive SeMet.	Safe at physiological concentrations, excessive intake may trigger toxicity or imbalance
Application	Dietary supplements, selenium-rich foods	Dietary supplements, selenium-rich foods	Precise selenium supplementation; Functional food/drug development goals	Disease diagnostic biomarkers; therapeutic targets

## Data Availability

No new data were created or analyzed in this study. Data sharing is not applicable to this article.
